# Parental feeding practices as a response to child appetitive traits in toddlerhood and early childhood: a discordant twin analysis of the Gemini cohort

**DOI:** 10.1186/s12966-023-01440-2

**Published:** 2023-04-04

**Authors:** Alice R. Kininmonth, Moritz Herle, Kristiane Tommerup, Emma Haycraft, Claire Farrow, Helen Croker, Abigail Pickard, Katie Edwards, Jacqueline Blissett, Clare Llewellyn

**Affiliations:** 1 Research Department of Behavioural Science and Health, Institute of Epidemiology and Health Care, University College London, London, UK; 2 Social, Genetic & Developmental Psychiatry Centre, Institute of Psychiatry, Psychology & Neuroscience, King’s College London, London, UK; 3 School of Sport, Exercise and Health Sciences, Loughborough University, Loughborough, UK; 4 School of Psychology & Institute of Health and Neurodevelopment, Aston University, Birmingham, UK; 5 World Cancer Research Fund International, London, UK

**Keywords:** Discordant twin, Discordance, Sibling, Eating behaviour, Child, Toddler, Feeding practices

## Abstract

**Background:**

Parental feeding practices (PFPs) have been implicated in the development of children’s eating behaviours. However, evidence suggests that feeding practices may also develop in response to their child’s weight or emerging appetitive traits. We used the twin design to test the hypothesis that parents develop their feeding practices partly in response to their child’s appetite.

**Methods:**

Data were from Gemini, a population-based cohort of 2402 British families with twins born in 2007. Psychometric measures of PFPs and appetite were completed by parents when their twins were 16-months and 5-years. Within-family analyses including all twins with available data in the sample (n = 1010–1858 pairs), examined if within-pair differences in PFPs were associated with differences in appetitive traits, controlling for differences in birth weight-SDS, early feeding method and child sex. In a subsample of twin pairs who were considerably discordant for appetitive traits by ≥ 1SD (n = 122–544 pairs), the direction and magnitude of within-pair differences in feeding practices was explored.

**Results:**

Within-family variation in parental feeding practices in toddlerhood and early childhood was low (discordance ranged from 0.1 to 6% of the sample), except for pressure to eat (toddlerhood: 19%; early childhood: 32%). Within-pair differences in all appetitive traits were associated with differential use of ‘pressure to eat’ at both 16-months and 5-years. In the subsample of twins most discordant for appetitive traits, parents used more pressure with the twin expressing lower food responsiveness, lower emotional overeating, lower food enjoyment, higher satiety responsiveness, slower speed of eating, higher emotional undereating and greater fussiness in toddlerhood and early childhood (p-values < 0.001). Effect sizes were small to large at 16-months (η^2^=0.02–0.09) and 5-years (η^2^=0.05–0.21).

**Conclusion:**

Parents rarely varied their feeding practices between twins in toddlerhood and early childhood, except for pressure. Parents exerted greater pressure on their twin who expressed a poorer appetite compared to their co-twin, suggesting that parents develop a pressuring feeding style when their child expresses a poorer appetite or lower interest in, and enthusiasm for, eating. These findings could be used to guide interventions seeking to support parents in feeding their children in a way that nurtures the development of healthy eating behaviours.

**Supplementary Information:**

The online version contains supplementary material available at 10.1186/s12966-023-01440-2.

## Introduction

Parents are often considered the ‘gatekeepers’ of their child’s food environment, especially during the preschool years, influencing the ‘what’, ‘when’ and ‘how’ of children’s eating through their feeding practices [1]. As such, parental feeding practices are a core component of a child’s food environment and have been implicated in the development of children’s food preferences [2], eating behaviours and weight [3, 4]. To date, nonresponsive feeding practices such as pressuring a child to eat (known as ‘pressure to eat’), restricting a child’s intake or access to food (termed ‘overt restriction’), and using food to reward or punish behaviour (termed ‘instrumental feeding’) or to soothe emotions (termed ‘emotional feeding’) have received considerable attention in the literature, and variation in the use of these feeding practices has been associated with variation in children’s appetite [5, 6] and weight [3]. Evidence from cross-sectional and longitudinal studies has shown associations between nonresponsive feeding practices and appetite traits that characterise a more avid eating behaviour (for example, higher food responsiveness, higher emotional overeating, and lower satiety responsiveness) in childhood. In particular, using food to reward or soothe emotions has been prospectively associated with increases in food responsiveness [7] and emotional overeating [8], while greater pressure to eat has been prospectively associated with slower speed of eating and higher food fussiness [9]. More responsive feeding practices, such as modelling of healthy eating and providing structure around what and when food is offered and available, have received less attention in relation to children’s eating behaviours, with more of a focus on how they link to children’s dietary intake [10].

While much of the literature to date has focussed on relationships between feeding and eating behaviour from parent to child [5, 6, 11, 12], there is an emerging body of prospective research examining the direction of associations from child to parent [8, 13]. This literature suggests that parents may develop or adapt their feeding practices in response to the characteristics of their child, such as their weight status or appetite [8, 13]. For example, one prospective study (n = 207) observed that parents of children who were fussier around food developed more pressuring or rewarding feeding practices over time, to try to coerce their child to eat [14]. Such prospective studies provide support for a child-responsive model of feeding behaviour.

One powerful design for testing the hypothesis that parents develop their feeding practices in response to their child’s appetitive traits is a discordant twin or sibling design. In a discordant twin design, it is proposed that a parent will *only* use different feeding practices with their twins (or siblings) if they are responding to different characteristics expressed by each child. Previous research using the twin design (n = 1013 twin pairs, 16-months-old) found that parents did indeed vary their feeding practices if their twin children varied in their fussiness around food, with mothers using more pressure and instrumental feeding with the fussier twin [15]. Similar findings were observed in a small sibling study (n = 80 families, 3-6-years-old), with parents using more nonresponsive feeding practices with the sibling who was fussier, ate more slowly, enjoyed food less and was less responsive to food [16]. Another study conducted in 69 same-sex twin pairs revealed that mothers used more restriction with the twin with a higher BMI z-score and who had poorer ability to compensate their caloric intake in a lab-based setting [17]. A core strength of twin and sibling studies is that they are able to examine the association between parental feeding practices and children’s appetite, controlling for all possible confounding environmental influences that are shared completely by twin pairs or siblings living in one family/household (e.g., socioeconomic status, parental weight status, etc.; for a detailed review see Pingault et al., 2018) [18].

To date, only a handful of studies have used twins or siblings to interrogate the nature of the relationship between parental feeding practices and child eating behaviour [15, 16, 19]. These studies have been limited in scope, focussing on only a few appetitive traits and feeding practices, and have been limited to one timepoint. Understanding the role of child appetite in shaping parental feeding practices is crucial for developing tailored interventions to optimise parental feeding practices. The current study used a large population-based sample of British families with twins to test the hypothesis that parents develop their feeding practices partly in response to their child’s individual appetite. Specifically, this study aimed to: (i) understand the extent to which the same parent varies their feeding practices between twin pairs in toddlerhood (16 months) and early childhood (5 years); (ii) determine whether variation in parental feeding practices is associated with within-pair differences in children’s appetitive traits; and (iii) understand the direction and magnitude of differences in feeding practices for twins considerably discordant for appetitive traits. We hypothesise that (aim i) there is considerable variation in parental feeding practices within families of twins in toddlerhood and early childhood; and that (aim ii) within-parent variation in feeding practices is positively associated with within-pair variation in appetitive traits; and (aim iii) parents will use more nonresponsive feeding practices with their twin who expresses a poorer appetite and lower interest in eating.

## Methods

### Sample

Participants were from the Gemini study, a longitudinal birth cohort of families with twins born in England and Wales between March and December 2007. In total, 2,402 families with monozygotic (identical) and dizygotic (non-identical) twins (n = 4804) consented to take part and completed baseline questionnaires when their children were a mean (± Standard Deviation [SD]) of 8.2 (± 2.2) months old. The recruitment of the sample has been described in detail elsewhere [20]. Data used in this study are from baseline, 16 months, and five years. Of the 2402 families who completed the baseline questionnaire, 1931 families (80.4%) completed the 16 months questionnaire, and 1087 families (45.3%) completed the five years questionnaire. The analysis sample comprised 1858 families at 16 months (3716 children; 1886 [50.8%] female) and 1010 families at 5 years (2020 children; 1037 [51.3%] female) (Table 1). The twins’ primary caregiver provided written informed consent for their family to participate in the study. Ethical approval was granted by the University College London Committee for the Ethics of non-National Health Service Human Research.

**Table 1:** Characteristics of analysis sample at 16 months and 5 years

	Sample at 16 months (n = 1858 families; 3716 children)	Sample at 5 years (n = 1010 families; 2020 children)
**Child characteristics**	**Mean (SD) or n (%)**	**Mean (SD) or n (%)**
Sex (female)	1886 (50.8)	1037 (51.3)
Birth weight (kg)	2.47 (0.54)	2.46 (0.54)
Birth weight SDS	-0.55 (0.93)	-0.58 (0.92)
Age at questionnaire completion (twins)	15.81 (1.14)	5.15 (0.13)
Gestational age (weeks)	36.22 (2.46)	36.26 (2.44)
Early feeding method (mostly breastfed)	1654 (44.5)	984 (48.7)
Weight SDS at 16 months^1^	-0.08 (1.09)	-0.07 (1.08)
BMI-SDS at 5 years^2^	-	-0.23 (1.10)
**Maternal characteristics**		
Maternal age at twins’ birth (years)	33.33 (5.04)	33.84 (4.75)
**Appetitive traits, mean (SD)**		
Food responsiveness	2.27 (0.76)	2.37 (0.75)
Emotional overeating	1.65 (0.59)	1.57 (0.51)
Enjoyment of food	4.18 (0.62)	3.89 (0.68)
Satiety responsiveness	2.68 (0.62)	2.85 (0.62)
Slowness in eating	2.49 (0.65)	2.82 (0.77)
Food fussiness	2.18 (0.70)	2.77 (0.83)
Emotional undereating	-	2.68 (0.84)
**Parental feeding practices, mean (SD)**		
Emotional feeding	2.05 (0.72)	1.70 (0.54)
Instrumental feeding	1.35 (0.47)	2.33 (0.63)
Pressure to eat	2.24 (0.73)	2.75 (0.67)
Restriction	5.21 (1.25)	5.14 (1.10)
Covert restriction	3.07 (0.92)	2.98 (0.80)
Modelling of healthy eating	3.41 (0.84)	3.71 (0.72)
Encouragement to eat healthy foods	4.07 (0.61)	4.13 (0.53)
Parent control over meals/snacks	4.45 (0.48)	4.15 (0.44)
Monitoring	3.62 (1.02)	3.55 (0.91)
**Appetitive traits**	**Number of pairs with a difference score > 0** **n (% of sample)**	
	**16 months**	**5 years**
Food responsiveness	731 (39.3)	487 (48.2)
Emotional overeating	194 (10.4)	123 (12.2)
Enjoyment of food	705 (37.9)	452 (44.8)
Satiety responsiveness	858 (46.2)	595 (58.9)
Slowness in eating	835 (44.9)	622 (61.6)
Food fussiness	918 (49.4)	644 (63.8)
Emotional undereating	-	274 (27.1)
**Parental feeding practices**	**16 months**	**5 years**
Emotional feeding	43 (2.3)	19 (1.9)
Instrumental feeding	51 (2.7)	61 (6.0)
Pressure to eat	355 (19.1)	324 (32.1)
Restriction	36 (1.9)	36 (3.6)
Modelling of healthy eating	3 (0.2)	1 (0.1)
Covert restriction	5 (0.3)	5 (0.5)
Encouragement to eat healthy foods	13 (0.7)	37 (3.7)
Control over meals/snacks	19 (1.0)	25 (2.5)
Monitoring	6 (0.3)	14 (1.4)

^1^Missing data for 1653 children from 16 months sample, 820 children from 5 years sample.

^2^Missing data for 1261 children

## Measures

### Parental feeding practices

Nine Parental feeding practices (PFPs) were reported by the primary caregiver when their children were 16 months and 5 years old [21–24]. The nine scales included four nonresponsive (Instrumental feeding, Emotional feeding, Pressure to eat, Restriction) and five responsive PFPs (Parent control, Monitoring, Encouragement to eat nutritious foods, Modelling, Covert restriction). ‘Instrumental feeding’ measures caregivers’ use of food as a contingency for healthy food consumption or good behaviour (4 items; e.g., *‘I use puddings as a bribe to get my child to eat his/her main course’; 16 months:α = 0.50, 5 years:α = 0.68*) [21]. ‘Emotional feeding’ measures caregivers’ use of food to manage or control a child’s negative emotions (5 items; e.g. *‘I give my child something to eat to make him/her feel better when s/he is feeling upset’; 16 months:α = 0.85, 5 years:α = 0.80*) [21]. The ‘pressure to eat’ scale measures caregivers’ attempts to coerce the child to eat more (5 items; e.g. *‘My child should always eat all of the food I give him/her’; 16 months:α = 0.65, 5 years:α = 0.64*) [22]. ‘Restriction’ was measured using a scale specifically designed to measure parental tendency to limit a child’s access to and portion sizes of sugary and high fat foods (4 items, e.g. ‘*I limit my child’s access to sugary foods’; 16 months:α = 0.86, 5 years:α = 0.90*) [25]. The ‘Parent control’ scale examines the extent to which caregivers exert control over what their child eats at meals and snacks, and when they eat (5 items; e.g. *‘I let my child decide when s/he would like to have his/her meal’*) [21]. ‘Encouragement to eat’ assesses caregivers’ use of positive reinforcement to encourage their child to eat food (such as praise for trying a new food), particularly healthy foods (5 items; e.g. *‘I encourage my child to eat a wide variety of foods’; 16 months:α = 0.59, 5 years:α = 0.63*) [21]. ‘Monitoring’ assesses the extent to which caregivers keep track of their child’s high fat/sugary food consumption while in their own or others’ care (3 items; e.g. *‘I keep track of the high fat foods that my child eats’; 16 months:α = 0.72, 5 years:α = 0.73*) [22]. ‘Modelling’ assesses the extent to which caregivers model healthy eating to their children (4 items; e.g. *‘I model healthy eating for my child by eating healthy foods myself’; 16 months:α = 0.80, 5 years:α = 0.80*) [23]. ‘Covert restriction’ measures the extent to which parents restrict their child’s access to foods, supposedly without their child knowing (4 items; e.g. *‘I avoid buying unhealthy foods and bringing them into the house’; 16 months:α = 0.69, 5 years:α = 0.71*) [24]. All items were rated using a five-point Likert scale from ‘never’ [1] to ‘always’ [5], except the restriction scale which was measured on a 7-point Likert scale from not at all [1] to strictly [7]. A mean score was calculated for each of the scales for each twin if responses were available for most items within a scale (e.g., If participants had completed items for at least 2/3 items for monitoring, at least 3/4 items for modelling, restriction, covert restriction, and at least 3/5 items for remaining scales). All measures were validated in comparable populations, except for the restriction scale [21–24].

### Child eating behaviour

Child appetite was assessed at five years using the Children’s Eating Behaviour Questionnaire (CEBQ) [26] and at 16 months using the CEBQ-T (toddler version of the CEBQ) [27]. The CEBQ is a validated parent-reported psychometric measure of eight appetitive traits (seven eating behaviour traits and one drinking behaviour trait), which consists of 35 items, rated using a 5-point Likert scale (1 = Never to 5 = Always) [26, 28]. Food Responsiveness (FR) measures a child’s drive to eat in response to external food cues (5 items, e.g. *‘Given the choice, my child would eat most of the time’* 16 months:α = 0.76, 5 years:α = 0.81). Enjoyment of Food (EF) assesses a child’s subjective pleasure from eating (4 items, e.g. ‘My child loves food’; 16 months:α = 0.85, 5 years:α = 0.86). Emotional Overeating (EOE; 4 items, e.g. ‘My child eats more when worried’; 16 months:α = 0.82, 5 years:α = 0.77) and Emotional Undereating (EUE; 4 items, e.g. ‘*My child eats less when s/he is tired’; 5 years*: α = 0.77*)* assess the extent to which a child eats (more or less) in response to emotional stressors. Satiety Responsiveness (SR) measures a child’s sensitivity to internal cues of ‘fullness’ (5 items, e.g. *‘My child gets full up easily’; 16 months:α = 0.75, 5 years:α = 0.76*). Slowness in Eating (SE) refers to the speed of meal consumption (4 items, e.g. *‘My child eats slowly’; 16 months:α = 0.66, 5 years:α = 0.79*). Food Fussiness (FF) examines a child’s pickiness about the flavour and texture of foods they are willing to eat (6 items, e.g. ‘My child refuses new foods at first’; 16 months:α = 0.87, 5 years:α = 0.91). A mean score was calculated for each subscale for participants who had completed the majority of items for that scale (3/4 for EOE, EUE, EF, SE, 3/5 for FR, SR, 4/6 for FF). EUE was only measured at 5 years, it is not included in the CEBQ-T as mothers reported during the piloting of the questionnaire that their toddlers did not engage in this behaviour [27]. Desire to drink was not examined as this is a drinking behaviour trait and the focus was on eating behaviour traits.

## Covariates

Mothers reported the gestational age of the twins at delivery (weeks) and each child’s sex. The feeding method used in the first three months of life was indicated by the mother using the following response options: “exclusive breast feeding”, “mostly breastfed, some bottle”, “equal breast and bottle feeding”, “mostly bottle feeding, some breastfeeding”, “almost entirely bottle feeding” and “entirely bottle”. Responses were dichotomised into 1 = Mostly breastfed (“entirely, mostly or equally breastfed for 3 months”) or 0 = mostly bottle-fed (“entirely or mostly bottle-fed for 3 months”).

Primary caregivers consulted their child’s health records (completed by health professionals but held by the mother) when reporting birthweight and any subsequent weight measurements available at completion of the baseline (8 months), 16 months and 5 years questionnaires. Electronic weighing scales and height charts were sent to all families when the twins were aged two years to collect parent-reported height and weight measurements every 3 months. Weight (kg) data was converted into standard deviation scores (SDS) for child weight (Weight-SDS) at 16 months and body mass index (BMI-SDS) at 5 years using the UK 1990 British growth reference data [29], adjusting for age, sex, and gestational age.

### Statistical analysis

### Characterising twin pair discordance in parental feeding practices and appetite

Within-family differences between twin pairs for the seven eating behaviour appetitive traits and nine parental feeding practices were calculated by subtracting scores for Twin 2 (second born) from Twin 1 (first born) using a similar method to previous research [15, 16]. Table 1 outlines the number of twin pairs with a difference score > 0 for the appetitive traits and feeding practices.

### Analyses using the whole sample

Within-pair differences in each appetitive trait, for all twins with complete data at each age, were entered into a linear regression model (continuous independent variable) to determine if this was associated with within-pair differences in each feeding practices (continuous dependent variable). Separate models were run for differences in each appetitive trait with differences in each feeding practice. The models controlled for differences between twins in birth weight SDS, early feeding method and sex. As a sensitivity analysis, models were additionally adjusted for differences in weight-SDS at 16 months (for analysis at 16 months) or BMI-SDS at 5 years (for analysis at 5 years).

### Analyses using a subsample of twins discordant for appetitive traits

Repeated measures analysis of covariance were used to explore the magnitude of differences in feeding practices for a subsample of twins who were considerably discordant in their appetitive traits in toddlerhood (16 months) and early childhood (5 years). These analyses were used to explore differences in parental feeding practices between twin pairs who were discordant for each appetitive trait (for example, between the more food responsive twin and less food responsive twin). ‘Discordant twins’ were defined as twin pairs who had a difference score ≥ 1 standard deviation (SD) of the difference score for that appetitive trait. For example, for food responsiveness at 16 months this equated to a difference score ≥ 0.43 between twin pairs (see **Table**S2 for further details). The alpha level was 0.01 for all analyses.

## Results

The characteristics of the analysis samples at 16 months and 5 years are shown in Table 1. Compared to the baseline Gemini sample (n = 2402 families), primary caregivers in this subsample were significantly older at their twins’ birth (16 months: 33.34 years and 5 years: 33.83 years vs. baseline: 32.94) and had a significantly lower BMI (24.64 vs. 25.10), although the size of the differences was small. As shown in Table 1, despite considerable discordance in child appetitive traits at 16 months and 5 years, for the majority of feeding practices only a few parents varied their feeding practices across their two twins (rates ranged from 0.1 to 6% of the sample). The exception, however, was pressure to eat, which showed considerable within-parent variation among twin pairs; 19.1% of parents varied the amount of pressure they exerted on their two twins to eat at 16 months, rising to 32.1% of parents at 5 years.

### Within-parent differences in pressure to eat and within-pair differences in children’s appetite using the whole sample

Differences in parental ‘pressure to eat’ between two twins in a pair were significantly and positively associated with differences in all appetitive traits at both 16 months and 5 years (all p’s < 0.001; Table 2). In other words, a parent varied their feeding practices more when their two twin children had bigger differences in their appetite, in toddlerhood and early childhood. Effect sizes for all associations were small to moderate.

**Table 2:** Within-twin differences in ’pressure to eat’ and differences in appetitive traits at 16 months (n = 1858 twin pairs) and 5 years (n = 1010 twin pairs)

Differences in appetitive traits	Differences in Pressure to eat					
	16 months			5 years		
	B ± SE	*β* ± SE	P value	B ± SE	*β* ± SE	*p value*
Food responsiveness	**0.22 (0.01)**	**0.42 (0.02)**	**< 0.001**	**0.31 (0.03)**	**0.49 (0.05)**	**< 0.001**
Emotional overeating	**0.26 (0.03)**	**0.22 (0.02)**	**< 0.001**	**0.63 (0.11)**	**0.31 (0.06)**	**< 0.001**
Enjoyment of food	**0.29 (0.01)**	**0.53 (0.02)**	**< 0.001**	**0.37 (0.03)**	**0.62 (0.05)**	**< 0.001**
Satiety responsiveness	**0.32 (0.01)**	**0.53 (0.02)**	**< 0.001**	**0.44 (0.03)**	**0.67 (0.05)**	**< 0.001**
Slowness in eating	**0.16 (0.01)**	**0.34 (0.02)**	**< 0.001**	**0.24 (0.03)**	**0.47 (0.05)**	**< 0.001**
Food fussiness	**0.17 (0.01)**	**0.36 (0.02)**	**< 0.001**	**0.24 (0.04)**	**0.35 (0.06)**	**< 0.001**
Emotional undereating^1^	-	-	-	**0.48 (0.04)**	**0.34 (0.03)**	**< 0.001**

B indicates unstandardised estimate, β indicates the standardised estimate

^1^Emotional undereating was only collected at 5 years.

### Differences in pressure to eat in the subsample of twins discordant for appetitive traits

**Fig. 1:**
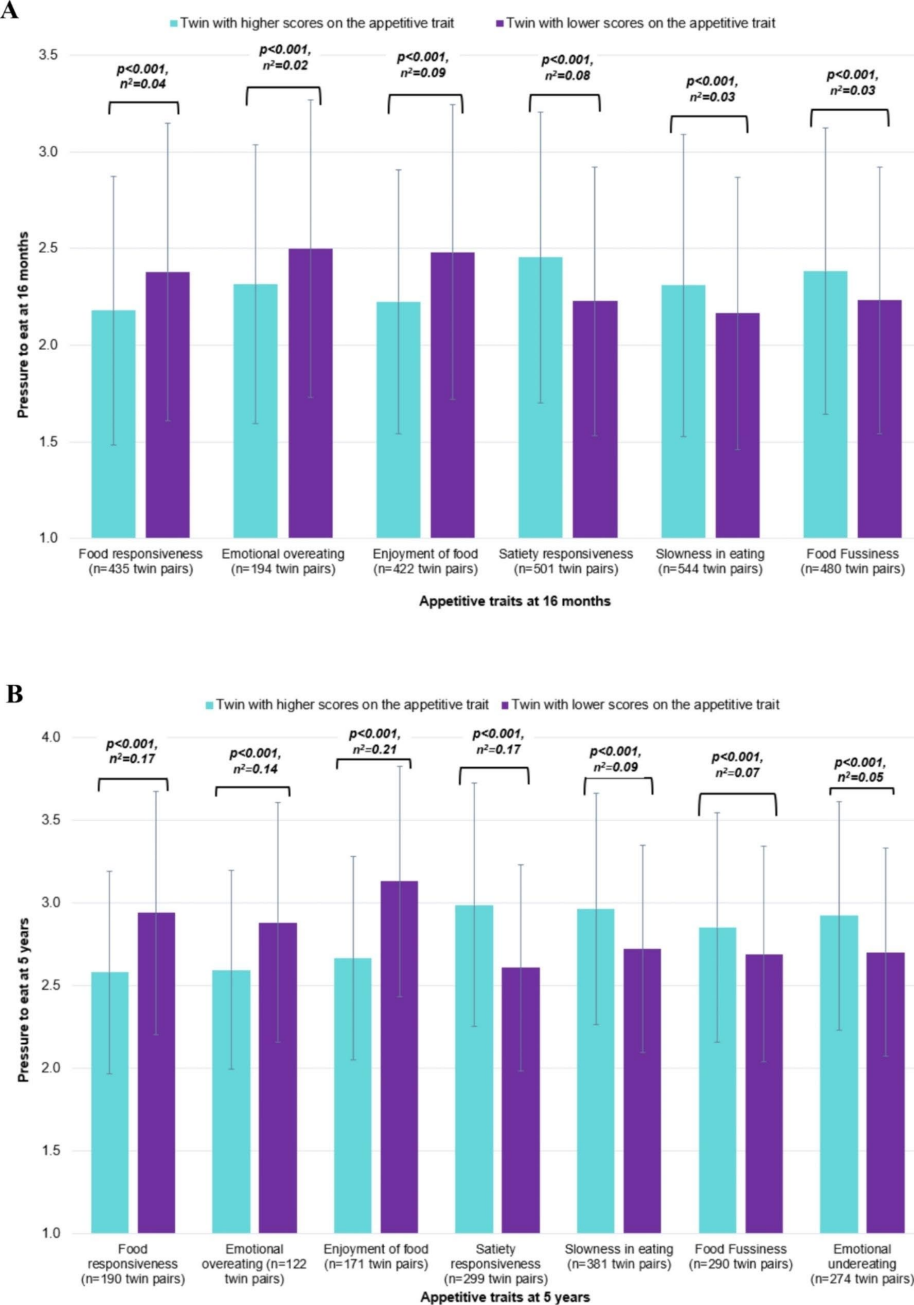
**A-B**: Differences in pressure to eat for twin pairs discordant in appetitive traits at 16 months and 5 years. Significance (p) and partial eta squared (ƞ^2^) effect size

The subsample of twins discordant for appetitive traits was used to explore the direction of differences in parental feeding practices and the magnitude of effect. These analyses indicated that in toddlerhood (16 months) and early childhood (5 years), parents exerted greater pressure to eat on their twin who expressed lower food responsiveness, lower enjoyment of food, lower emotional overeating tendencies, higher satiety responsiveness, slower speed of eating, higher emotional undereating and was fussier around food compared to their co-twin (Fig. 1). Effect sizes were small to large in magnitude, with ƞ^2^ ranging from 0.02 to 0.21. The effect sizes tended to increase in magnitude from toddlerhood to early childhood.

### Sensitivity analyses

Sensitivity analyses were conducted to adjust for differences in weight-SDS at 16 months (for analysis at 16 months) or BMI-SDS at 5 years (for analysis at 5 years). The findings for pressure to eat mirrored those observed in the main analysis, although the magnitudes of association were slightly attenuated when adjusting for child weight at measurement, but effects were still small to moderate in size (**Table**S1).

## Discussion

This is the most comprehensive study to date to use the discordant twin design to test the hypothesis that parental feeding practices are developed partly in response to children’s appetitive traits in toddlerhood (16 months) and early childhood (5 years of age). Our findings only partially supported this hypothesis. We observed that, despite considerable discordance in appetitive traits between twin pairs, parents did not vary their feeding practices between twin pairs for most feeding practices. The exception to this was pressure to eat for which considerable discordance in its use was observed. For pressure to eat, our findings supported the hypothesis that parents develop this feeding practice partly in response to their two twins expressing different appetites. In the analyses of the whole sample, we observed that within-parent variation in pressure to eat was significantly and positively associated with within-pair differences in appetitive traits in toddlerhood and early childhood – i.e. the extent to which a parent treated their two children differently with regard to pressuring them to eat, depended on how different their twin children were in their appetites. In the subsample of twin pairs who were considerably discordant for appetite, parents exerted more pressure to eat on their twin who expressed a poorer appetite and a lower interest in, and enthusiasm for, eating (characterised by lower food responsiveness, lower enjoyment of food, lower emotional overeating tendencies, more sensitivity to satiety cues, slower eating, higher emotional undereating and more fussiness around food) in both toddlerhood and early childhood. The findings were largely unchanged by adjustment for child weight, indicating that the parental pressure-appetite relationship does not simply reflect parental concern around child weight. These findings indicate that parents tend to pressure their children to eat to differing extents, and that this feeding practice is developed partly in response to a child’s emerging appetite. However, the concordance for most feeding practices suggests that, in this sample of twins, most feeding practices are parent-driven rather than child-driven.

Our findings support and extend previous twin and sibling research [15, 16], with one sibling study conducted in a sample of UK mothers and their 3–6 year old children observed that mothers used more pressure with their child who was fussier around food, enjoyed food less, ate slower, was more responsive to satiety cues and less food responsive [16]. Previous evidence has suggested that pressuring feeding practices may manifest in response to parents’ concerns about their child’s weight status [30, 31] or the adequacy of their child’s dietary intake [32]. This has been evidence in previous research using the twin design which revealed that mothers used more pressure to eat with their twin who had a lower weight [17]. Our findings highlight that greater pressure may also occur in response to the appetitive traits expressed by their child, with parents using more pressure with their twin who was less food responsive, had lower enjoyment of food, lower emotional overeating tendencies, had higher satiety responsiveness and a slower speed of eating, higher emotional undereating and was fussier around food compared to their co-twin. Although often well-intentioned, pressuring feeding practices may have a detrimental impact on children’s eating behaviours, with prospective studies reporting increases in fussiness around food [9], slower speed of eating [13], greater anxiety and lower intake of food at mealtimes [33, 34]. It is evident that pressuring feeding practices have a detrimental impact on children’s appetite and weight [33–37]. Therefore, future research needs to focus on providing parents with alternative approaches to feeding a child who expresses a poorer appetite and a lower interest in food, to support the development of healthy eating patterns. In addition, support needs to be provided to parents to help minimise their fear and anxiety around their child expressing these appetite traits.

Despite considerable discordance in appetitive traits between twin pairs, rates of discordance for all feeding practices, except for pressure to eat, were quite low. This suggests that in toddlerhood and early childhood, contrary to our hypothesis, feeding practices are not child-responsive – rather they reflect more general approaches to parenting and feeding. The low discordance for certain feeding practices (e.g., monitoring, modelling, covert restriction, encouragement to eat nutritious foods) may reflect the fact that these are feeding practices that typically cannot be varied by one parent, particularly within the same eating occasion. For example, it would be difficult for a parent to model healthy eating to differing extents to two twins within the same household, without actively separating the twins during all eating occasions. In contrast, it is more feasible to pressure one twin to a greater extent than the other twin in the same eating occasion. This has also been suggested in previous research [38]. However, previous research conducted in 69 same-sex twin pairs revealed that mothers used more restriction with their twin who had a higher BMI z-score and who expressed poorer ability to compensate their caloric intake in a lab-based setting compared to their co-twin [17]. Differences in restrictive feeding practices were also observed in a small sibling study which revealed that parents used more restriction with the child who was fussier [16]. In contrast, this was not observed in our larger sample. Our findings are encouraging in that they suggest that most parental feeding practices are parent-driven, except for pressure to eat. As most feeding practices do not appear to vary significantly in response to differences in child appetitive traits, public health campaigns could target parental motivators for the use of specific feeding practices to facilitate change in parental feeding practices. However, more targeted or individual level interventions may still be needed for families where children have clinically significant differences or who have appetitive traits that characterise a particularly avid (e.g. 5) or poor appetite (e.g. 1).

## Strengths and limitations

Strengths of the current study include the large sample size and the use of psychometric measures of parental feeding practices and child appetitive traits. In addition, the twin design removes confounding from all environmental factors that are shared completely by twin pairs, thus providing powerful evidence that pressuring feeding practices are partly a response to differences in appetite expressed by each child. However, there are limitations to this study that should be acknowledged. Firstly, the measures of parental feeding practices and children’s appetite were parent-reported and subjective in nature, thus may be susceptible to desirability biases which may introduce measurement error. However, good correspondence has been shown with more objective measures of eating behaviour [28]. Furthermore, some of the measures of PFPs, such as pressure to eat and encouragement to eat, had a Cronbach’s alpha below 0.70, indicating that they were not that reliable. Secondly, the sample comprised a larger proportion of mid-high SES families and the majority identified as White-British, limiting generalizability of the findings to families from more ethnically or socioeconomically diverse backgrounds [39]. More research is needed in large ethnically and socioeconomically diverse populations to clarify these findings. Lastly, although the twin design has many advantages, parents may feed twins differently to singletons. Compared to singletons, twins tend to be born earlier, and have a lower birth weight so experience ‘catch up’ growth [40]. However, research has shown that twins do not differ from singletons on various physical and behavioural traits later in life such as alcohol consumption and blood pressure [41, 42]. There is no evidence to suggest that the relationship between parental feeding practices and appetite would be different in a twin sample. Finally, it is important to acknowledge that creating difference scores between twin pairs or siblings does not allow us to distinguish between those twins who score particularly high or low on a scale compared those who score average for example, a child who scores 5 on a subscale may have the same difference score as a child who scores 2.5 on a subscale i.e. they have a difference score of 0.75 with their co-twin, but those who have appetitive traits that characterise a particularly avid (e.g. 5) or poor appetite (e.g. 1) may impact the extent to which parents vary certain feeding practices between children. Furthermore, at present we cannot discern what constitutes a clinically meaningful difference score between twins or siblings. Future research should aim to establish a clinically meaningful difference score between children within the same family.

## Conclusions

This is the most comprehensive study to use the twin design to test the hypothesis that parental feeding practices are developed partly in response to children’s appetitive traits in toddlerhood and early childhood. The findings revealed that for most feeding practices parents did not vary their feeding practices between their two twins. The exception to this was pressure to eat, for which considerable discordance was observed. Parents exerted greater pressure on the twin who expressed a poorer appetite and a lower interest in food and eating in both toddlerhood and early childhood. These findings suggest that parents adapt their pressuring feeding practices partly in response to the appetitive traits expressed by their children. Overall, however, our findings indicated that most feeding practices seem to be parent-driven behaviours, rather than a response to their child’s unique characteristics. These findings could be used to develop guidance to support parents around appropriate feeding practices to facilitate the development of healthy eating behaviours.

## Electronic supplementary material

Below is the link to the electronic supplementary material.

Supplementary Material 1: STROBE Statement - checklist of items that should be included in reports of observational studies

Supplementary Material 2: Table S1 and Table S2

## Data Availability

The datasets used and/or analysed during the current study are available from the corresponding author on reasonable request.

## Abbreviations

PFPs: Parental Feeding Practices
SD: Standard Deviation
CEBQ: Child Eating Behaviour Questionnaire
CEBQ-T: Child Eating Behaviour Questionnaire - Toddler
SDS: Standard Deviation Scores
FR: Food Responsiveness
EF: Enjoyment of food
EOE: Emotional overeating
SR: Satiety Responsiveness
SE: Slowness in eating
FF: Food Fussiness
BMI-SDS: Body Mass Index Standard Deviation Scores
