# Supplementary material for: Parental feeding practices as a response to child appetitive traits in toddlerhood and early childhood: a discordant twin analysis of the Gemini cohort
Source: Int J Behav Nutr Phys Act. 2023 Apr 4;20:39. doi: 10.1186/s12966-023-01440-2 (PMC10074660; doi:10.1186/s12966-023-01440-2)
Supplement: Supplementary file 2 — Supplementary Material 2: Table S1 and Table S2 [file 12966_2023_1440_MOESM2_ESM.docx]

**Supplementary material**

**Table S1:** Sensitivity analysis: Within-twin differences in appetitive traits and parental feeding practices. Analyses controlling for differences in weight-SDS at 16 months (n=1022) or BMI-SDS at 5 years (n=376).

| **Differences in appetite** | **Differences in Pressure to eat** | | | | | |
| --- | --- | --- | --- | --- | --- | --- |
|  | **16 months** | |  | **5 years** | |  |
|  | **B ± SE** | ***β* ± SE** | **p value** | **B ± SE** | ***β* ± SE** | p value |
| Food Responsiveness | **0.20 (0.02)** | **0.37 (0.03)** | **<0.001** | **0.25 (0.03)** | **0.39 (0.05)** | **<0.001** |
| Emotional Overeating | **0.24 (0.03)** | **0.21 (0.03)** | **<0.001** | **0.57 (0.11)** | **0.26 (0.05)** | **<0.001** |
| Enjoyment of food | **0.27 (0.02)** | **0.48 (0.03)** | **<0.001** | **0.30 (0.03)** | **0.49 (0.04)** | **<0.001** |
| Satiety responsiveness | **0.28 (0.02)** | **0.47 (0.03)** | **<0.001** | **0.35 (0.03)** | **0.57 (0.04)** | **<0.001** |
| Slowness in eating | **0.15 (0.01)** | **0.33 (0.03)** | **<0.001** | **0.20 (0.02)** | **0.46 (0.05)** | **<0.001** |
| Food fussiness | **0.13 (0.01)** | **0.28 (0.03)** | **<0.001** | **0.10 (0.02)** | **0.20 (0.05)** | **<0.001** |
| Emotional undereating^1^ | - | - | - | **0.44 (0.06)** | **0.33 (0.05)** | **<0.001** |
| B indicates unstandardised estimate, β indicates the standardised estimate  ^1^Emotional undereating was only collected at 5 years. | | | | | | |

**Table S2:** Mean (SD) of the discordance scores for each appetitive trait at 16 months (n=1858) and 5 years (n=1010). Discordant twins were defined as twin pairs who had a difference score greater than or equal to one standard deviation of the difference score for that appetitive trait.

| Appetitive traits | Mean (SD) discordance scores | |
| --- | --- | --- |
|  | **16 months** | **5 years** |
| Food responsiveness | 0.25 (0.43) | 0.30 (0.52) |
| Emotional overeating | 0.06 (0.20) | 0.05 (0.16) |
| Enjoyment of food | 0.23 (0.43) | 0.30 (0.52) |
| Satiety responsiveness | 0.24 (0.38) | 0.39 (0.52) |
| Slowness in eating | 0.31 (0.50) | 0.60 (0.73) |
| Food fussiness | 0.31 (0.48) | 0.54 (0.68) |
| Emotional undereating | - | 0.11 (0.23) |
